# The Von Restorff effect in the Brazilian version of the Rey Auditory Verbal Learning Test in an elderly population

**DOI:** 10.1590/1980-57642018dn13-010010

**Published:** 2019

**Authors:** Gabriel Lima, Alina Teldeschi, Natália Oliveira, Camila Bernardes, Cláudia Drummond, Naima Assunção, Tiago Bortolini, Paulo Mattos

**Affiliations:** 1 Centro de Neuropsicologia Aplicada - Institute D’Or of Research and Education (IDOR), Rio de Janeiro, RJ, Brazil.; 2 Memory Clinic - Institute D’Or of Research and Education, Rio de Janeiro, RJ, Brazil.; 3 Federal University of Rio de Janeiro (UFRJ), Rio de Janeiro, RJ, Brazil.; 4 Institute D’Or of Research and Education (IDOR), Rio de Janeiro, RJ, Brazil.

**Keywords:** memory, verbal learning, neuropsychological tests, memory disorders, dementia, memória, aprendizagem verbal, testes neuropsicológica

## Abstract

The Von Restorff (isolation) effect refers to a stimulus that is more likely to be remembered amongst other stimuli in memory tasks. It has been demonstrated with different age ranges and methodologies.

**Objective:**

To investigate: a) the presence of the isolation effect in elders tested with the new Brazilian Portuguese version of the Rey Auditory Verbal Learning Task (RAVLT) in which a word with potential emotional weight (mother) was introduced; b) whether isolation effects persist in memory disorders of different degrees of severity (Mild Cognitive Impairment [MCI]; Alzheimer’s Dementia [AD]).

**Methods:**

The RAVLT was administered to 287 consecutive volunteers. Individuals underwent medical and neuropsychological evaluation and were further sub-grouped into normal controls (n=114), MCI (n=87) and AD (n=86) patients. One-way analysis of variance (ANOVA) and Chi-squared tests were performed. *Post-hoc* Tukey analysis was conducted to assess significance of group differences.

**Results:**

There were significant group effects on the learning curve. A W-shape - instead of the classical U-shape - was found for the serial position curve in all groups.

**Conclusion:**

The new Brazilian version of the RAVLT exhibited the Von Restorff effect, where this phenomenon was evident not only in older adults but also patients with MCI and AD, providing further psychometric measures for inter-group analyses.

## RESUMO.

Efeito de von Restorff (efeito de isolamento) refere-se ao estímulo que é mais facilmente lembrado em relação a outros em tarefas de memória. Tal efeito foi demonstrado em diferentes faixas etárias com diferentes metodologias.

**Objetivo:**

Investigar: a) presença do efeito de isolamento numa população idosa avaliada com a nova versão brasileira do Teste de Aprendizagem Auditivo-Verbal de Rey (RAVLT), em que uma palavra com potencial carga emocional (‘mãe’) foi introduzida; b) se o efeito de isolamento persiste em distúrbios de memória de diferentes gravidades (Comprometimento Cognitivo Leve [MCI]; Demência de Alzheimer [AD]).

**Métodos:**

RAVLT foi aplicado em 287 voluntários. Indivíduos participaram de avaliações médica e neuropsicológica e foram posteriormente agrupados em controles normais (n=114), MCI (n=87) e DA (n=86). Análises de variância e testes de Chi-quadrado foram realizados. Análises *post-hoc* foram conduzidas para avaliar diferenças de características entre os grupos.

**Resultados:**

Observaram-se efeitos significativos de grupo na curva de aprendizagem. Todos os grupos apresentaram curva de posição serial em formato W - invés do clássico formato em U.

**Conclusão:**

A nova versão brasileira do RAVLT evidenciou um efeito de von Restorff. Tal fenômeno foi visto não apenas em idosos, mas também em pacientes com MCI e AD, fornecendo medidas psicométricas adicionais para análises de diferenças intergrupais.

When multiple homogeneous stimuli are presented during a memory task, the stimulus that differs from the rest is more likely to be remembered, a phenomenon called the von Restorff effect or “isolation effect”, named after the German psychiatrist and pediatrician Hedwig von Restorff (1906-1962).^1^

Several theories attempting to explain why the distinctive item is easier to recall have been proposed. First, a “surprise effect” was suggested, where the distinctiveness of a feature caused an increase in the individual’s attention levels, thus receiving enhanced processing at input.^2^ According to this theory, the contextual incongruity of the “isolated item” is what leads to the differential attention to this item. Based on this assumption, an isolation effect would not be expected if the isolated item were presented *prior* to a list of consistent items, a theory that contradicts Von Restorff’s initial findings.^3^

To best illustrate the role of attention and extra processing in supporting the isolation effect, a three-factor framework was developed,^4^ which was further expanded.^5^ The first factor of this framework explains that arousing stimuli can automatically attract attention and are easily remembered independent of context. The second and third factors explains how task demands can influence the selection of relevant stimulus contrasts and address the extent to which sample features are useful in the test. Based on this framework, memory for unusual events are determined by the combination of all three factors.^6^

Many isolation paradigms have been developed since the phenomenon was first described: the isolated item can be different from others by differing physically (a different color or font size), semantically (meaningfulness or category) or even a taboo word presented in a list of neutral words.^7^ In all cases, people demonstrate superior recall of these items when isolated.^3^^,^ 8 ^,^ 9 Therefore, the Von Restorff effect has been demonstrated numerous times in children and adults and, although it was first considered absent in elders,^10^ subsequent studies showed it is also present in older ages, albeit with a smaller effect size.^7^^,^ 11 Other studies have also reported the effect in a healthy elderly population, but failed to observe the effect in Alzheimer’s Disease (AD) patients.^13^ In all these studies, the paradigm used was the free recall of word lists, a task highly susceptible to isolation effects.^14^ However, the word lists were visually presented, and the isolated stimuli was mainly “physically” different from others,^7^^,^ 10 ^-^ 12 requiring distinctive processing and encoding.^13^^,^ 14

The Rey Auditory Verbal Learning Test (RAVLT) is a task that requires free recall of a word list and is commonly used to assess episodic memory impairment in clinical settings. The RAVLT version first translated for the Brazilian population^15^ disregarded the frequency of these words in Brazilian Portuguese language and the number of syllables in each word on the list.^16^ In a second paper, another translation of the word list was proposed,^16^ and remains the current version commercially available in Brazil. In this later version, the authors aimed to create a list of high-frequency (one-syllable and two-syllable) nouns and evaluated the performance of a local elderly population aged between 60 and 89 years. According to the authors, the rationale for including high frequency words on the list was that the test could be used to evaluate individuals from different socioeconomic groups and educational backgrounds.^16^

Although the Von Restorff effect has been widely explored, studies involving memory-impaired elders due to clinical conditions such as Mild Cognitive Impairment (MCI) and AD remain scarce, and to our knowledge, there are no previous studies investigating the isolation effect in patients with MCI. Moreover, previously studies used mainly paradigms involving visual presentation of words and “physically” different stimulus, which depends on distinctive processing and encoding, comprising the presentation of several lists, hampering assessment of clinical populations with memory impairment. In fact, it was suggested that part of the AD group may have been unable to properly perform the test.^12^ In addition, due to recency and primacy effects, usually seen in such tasks, the number of recalled items according to their serial position (i.e. serial position curve) has a U-shape.^17^^-^ 21 Therefore, the presence of an isolation effect could disrupt the serial position curve shape.

In the present study, we investigated the presence of an isolation effect in the current Brazilian version of the RAVLT. We hypothesized that the word *‘MÃE’* (mother), introduced in the second version, could lead to a Von Restorff effect, as it is considered an emotional stimulus in paradigms used to assess emotional memory.^22^^,^ 23 Because this word is placed in the sixth position of the list and potentially carries an emotional weight (absent in all other words), we hypothesized that the serial recall position curve would approximate to a ‘W’-shape, instead of the classical ‘U’-shape commonly seen in RAVLT due to an isolation effect in a middle position. In addition, because only one study has explored this effect in clinical elderly patients, we investigated whether the isolation effect persisted in memory disorders with different degrees of severity. For this last objective, we investigated the performance of normal elders, as well as individuals with Mild Cognitive Impairment and Dementia.

## METHODS

### Participants

This is a cross-sectional study, part of a larger study on pathophysiological (biomarkers and neuroimaging), neuropsychological and clinical aspects of normal ageing and cognitive decline in a specialized clinic. Some individuals were referred by health professionals, while others were self-referred and all subgrouped into: a) control individuals (normal ageing); b) Mild Cognitive Impairment (MCI) patients; or c) Dementia patients. All participants provided written informed consent to participate in this study, which was approved by the Ethics Committee of the Instituto D’Or de Pesquisa e Ensino (IDOR), Rio de Janeiro city, Rio de Janeiro state, Brazil. Control individuals were either spouses (both sexes) without memory complaints who also volunteered or individuals with memory complaints who were considered cognitively normal after extensive evaluation (this last procedure aimed to make the control group less strict, rendering any group differences more conservative).

Exclusion criteria were sensory impairments, infectious diseases, psychiatric disorders, neurological disorders and unstable or severe clinical conditions. Low educational level (below 8 years) was also an exclusion criterion due to its impact on other neuropsychological measures (RAVLT is one of the few exceptions) which the larger study comprised. Diagnoses were made by board-certified psychiatrists and neurologists based on clinical, neuropsychological and phono-audiological data. MCI diagnoses were based on previously proposed criteria,^24^ but were restricted to the amnesic subtypes (single and multiple domain) because the aim was to investigate individuals with memory impairment. Diagnoses of dementia due to AD were based on the DSM-5;^25^ whereas dementias of other causes were excluded for the purposes of this study. Clinicians also used the NPI-C (Neuropsychiatric Inventory-Clinician rating scale) to evaluate neuropsychiatric symptoms frequently present in dementia.^26^

Two hundred and eighty-seven consecutive volunteers participated in this study (106 men and 181 women, aged from 60 to 89 years). Overall, individuals had a high educational level and most came from a high socioeconomic stratum according to CCEB (Brazilian Economic Classification Criteria) classification.^27^

The control group (*n*=114) comprised individuals with unimpaired performance on a comprehensive neuropsychological assessment encompassing several cognitive functions (attention, memory, visuoperceptual skills, executive functions and language), described in detail elsewhere.^28^^,^ 29 The amnestic MCI group (*N*=87) presented memory impairment without impact on their Activities of Daily Living, in contrast to AD patients in the dementia group (*N*=86). In order to control for depressive symptomatology, which may impair memory performance, the Geriatric Depression Scale (GDS) was applied.

### Instruments and measures

The Geriatric Depression Scale (GDS) is commonly used to detect the presence of depressive symptoms in elderly patients.^30^ Here, we used the reduced version (GDS-15), which is validated for the Brazilian population.

The current proposed Brazilian version of the RAVLT was used.^23^ The Rey Auditory Verbal Learning Test (RAVLT) is a 15-word list recall test and one of the most widely used tasks in clinical neuropsychology.^15^ The list is read aloud by the examiner five times, and each repetition is followed by an immediate recall by the examinee (A1-A5 - learning phase). After these five trials, the examiner presents a distractor list, followed by an immediate recall of this second 15-word list. The patient is then asked to recall the first 15-word list, without it being repeated (A6 - interference phase). Another free recall trial is performed after 20 or 30 minutes (A7 - delayed recall phase), followed by a recognition phase.^15^^,^ 16 For the purposes of the present study, only the learning step results were analyzed and included in the Von Restorff effect score, which was calculated as the number of times the Von Restorff effect word (mother) was repeated during these five trials.

### Statistical analysis

A 0.5 two-tailed significance threshold (α) was adopted for all statistical tests, performed using SPSS 20.0 software. The assumption of normality was checked for all variables according to the Shapiro-Wilk test. When the assumption could not be attained, *bootstrapping* with 5,000 samples was performed, as suggested by other authors.^31^

In order to compare demographic variables and depressive symptoms across groups, one-way analysis of variance (ANOVA’s) and Chi-squared tests were performed. Post-hoc Tukey analysis was conducted to assess significance of group differences. When the assumption of homogeneity of variances was broken, we chose to report *Welch’s F* as it tends to be robust in such cases.^32^

To evaluate overall performance and the presence of the isolation effect across groups, both one-way multivariate analysis of covariance (MANCOVA) and one-way analysis of covariance (ANCOVA) were performed. On MANCOVA, when the assumption of homogeneity of covariance matrices was unattainable, we chose to report Pillai’s Trace (*V*), because it is considered more robust when this assumption is broken in larger unequal sample sizes.^33^^,^ 34 In addition, size effects (partial eta-squared) were reported for each analysis of covariance and interpreted in accordance with the guidelines postulated by Cohen.^35^

## RESULTS

### Group characteristics

We found significant differences across groups for age [*Welch’s F*(2, 177)=17.60, p <.001] and years of education [*Welch’s F*(2, 175)=7.93, p <.001], as expected since MCI and AD are more prevalent in older ages and low educational levels are associated with pathological ageing. However, Tukey post-hoc analysis revealed no significant differences between MCI and AD group characteristics (*p* >.05). In addition, we found non-significant differences among all three groups for depressive symptoms [*F*(2, 284)=2.61, *p*=.08] and for gender in each group [χ^2^(2, N=287)=5.37, *p*=.07]. All group characteristics and between-group comparison results are reported in Table 1.

**Table 1:** Age, Education, GDS Score and Sex: one-way ANOVA and Chi-squared results among the three groups.

Groups		Control (*N* =114)		MCI (*N* =87)		AD (*N* =86)	*F*	*p*-value	*Post-hoc* Tukey analysis
Variables		Mean (SD)		Mean (SD)		Mean (SD)			
GDS Score		3.61 (3.14)		3.86 (2.97)		4.62 (3.36)	2.61	*p* >.05	-
Age		70.37 (6.04)		74.07 (6.18)		75.79 (7.64)	17.60	*p* < .001	Control < MCI** Control < AD**
Education (years)		14.56 (2.92)		13.45 (3.21)		12.73 (3.71)	7.93	*p* < .01	Control > MCI* Control > AD**
**Sex**		***N* (%)**		***N* (%)**		***N* (%)**	χ **^2^**	***p*-value**	**-**
Males		43 (37.7%)		39 (44.8%)		24 (27.9%)	5.37	*p* > .05	-
Females		71 (62.3%)		48 (55.2%)		62 (72.1%)			-

SD: standard deviation; GDS: Geriatric Depression Scale; MCI: Mild Cognitive Impairment; AD: Alzheimer's disease.

* *p* <.05;

** *p* <.001.

### Overall performance on the RAVLT

**Figure 1:**
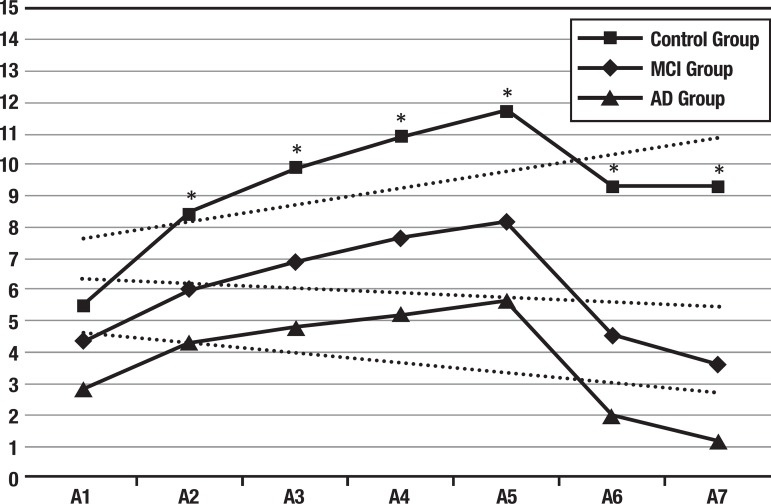
Performance curve for all three groups across RAVLT trials. Dotted lines show the linear tendency of each group serial recall curve. *p <.001. A1-A7: repetitions of list A from the RAVLT. MCI: Mild Cognitive Impairment. AD: Alzheimer’s disease.

The serial recall curves for each group are shown in Figure 1. A main group effect in the serial recall curve was observed [*V*=.74, *F*(14, 552)=23.60, *p* <.001, ηp^2^=.37], as well as a moderate effect of age [*V*=.06, *F*(7, 275)=2.46, *p* <.001, ηp^2^=.06], as expected. As shown in Table 2, the size effect for group increases after each trial (e.g. from A1 to A7). When each repetition was analyzed separately, small size effects were found for age in A1 [*F*(1, 281)=12.59, *p* <.001, ηp^2^=.04] and A6 [*F*(1, 281)=3.91, *p* <.05, ηp^2^=.014]. In addition, small size effects were found for educational level in A4 [*F*(1, 281)=4.01, *p* <.05, ηp^2^=.014], A5 [*F*(1, 281)=8.15, *p* <.01, ηp^2^=.03], A6 [*F*(1, 281)=3.92, *p* <.05, ηp^2^=.014] and A7 [*F*(1, 281)=8.02, *p* <.01, ηp^2^=.03].

**Table 2:** MANCOVA and ANCOVA results for group effects and comparison of performance across trials between groups.

Groups		Control (*N* =114)		MCI (*N* =87)		AD (*N* =86)	*F*	*p*-value	Size effect (ηp^2^)
Variables		Mean (SD)		Mean (SD)		Mean (SD)			
		One-way Analysis of Covariance							
Overall performance		-		-		-	23.60	*p* < .001.	.37
A1		5.47 (1.66)		4.34 (1.59)		2.79 (1.62)	44.86	*p* < .001.	.24
A2		8.41 (2.01)		6.02 (1.56)		4.29 (1.89)	99.02	*p* < .001.	.40
A3		9.91 (2.17)		6.87 (1.69)		4.77 (2.12)	127.84	*p* < .001.	.48
A4		10.90 (1.85)		7.62 (1.99)		5.21 (2.32)	153.43	*p* < .001.	.52
A5		11.70 (1.78)		8.18 (2.18)		5.63 (2.32)	167.51	*p* < .001.	.55
A6		9.26 (2.45)		4.52 (2.20)		1.94 (1.76)	228.69	*p* < .001.	.62
A7		9.28 (2.73)		3.61 (2.44)		1.16 (1.54)	260.27	*p* < .001.	.65

MCI: Mild Cognitive Impairment; AD: Alzheimer's disease.

### Serial position curve and the isolation effect

A main group effect was found on the serial position curve [*V*=.67, *F*(30, 536)=8.92, *p* <.001, ηp^2^=.33]. ANCOVA results for group effects on each word from the list, depicted in Table 3, also showed that although groups differed significantly from each other on almost every word, the highest size effect for group occurred for the Von Restorff word (mother). In addition, the number of times this word was repeated during the learning phase approximates to the number of times the words from the primacy and recency regions were repeated, although the same did not occur in the AD group.

**Table 3:** MANCOVA and ANCOVA results for group effects in the serial position curve and in each word from the list.

Groups		Control (*N* =114)		MCI (*N* =87)		AD (*N* =86)	*F*	*p*-value	Size effect (ηp^2^)
Variables		Mean (SD)		Mean (SD)		Mean (SD)			
		One-way Analysis of Covariance							
SPC		-		-		-	8.92	<.001	.33
BALÃO		4.46 (0.84)		3.57 (1.41)		2.59 (1.77)	36.26	<.001	.21
FLOR		3.76 (1.20)		2.66 (1.51)		1.66 (1.69)	42.81	<.001	.23
SALA		2.58 (1.31)		1.61 (1.49)		0.67 (1.14)	38.32	<.001	.21
BOCA		2.73 (1.51)		1.56 (1.58)		1.19 (1.50)	20.37	<.001	.13
CHUVA		2.75 (1.45)		1.43 (1.44)		1.12 (1.33)	34.53	<.001	.20
MÃE		3.83 (1.06)		2.72 (1.42)		1.53 (1.55)	56.53	<.001	.29
CIRCO		2.24 (1.38)		1.03 (1.39)		0.41 (0.85)	48.90	<.001	.26
PEIXE		2.25 (1.25)		1.09 (1.18)		0.56 (0.98)	53.48	<.001	.28
LUA		2.53 (1.37)		1.68 (1.51)		0.88 (1.32)	23.09	<.001	.14
CORPO		2.07 (1.47)		1.41 (1.57)		0.63 (1.15)	17.55	<.001	.11
CESTA		2.39 (1.57)		1.76 (1.67)		1.05 (1.57)	13.06	<.001	.09
LÁPIS		3.10 (1.36)		1.89 (1.57)		1.15 (1.40)	33.85	<.001	.19
MESA		3.45 (1.43)		2.62 (1.49)		2.14 (1.74)	10.94	<.001	.07
CHAPÉU		3.96 (1.24)		4.02 (1.11)		3.63 (1.62)	1.89	.15	.01
MILHO		4.29 (1.06)		4.07 (1.15)		3.50 (1.49)	5.92	<.001	.04

Furthermore, there was a significant effect of educational level on the serial position curve [*V*=.10, *F*(15, 267)=1.99, *p* <.05, ηp^2^=.10]. ANCOVA results for education effects on each word from the list showed a small size effect for the isolated word (mother) [F(1, 281)=3.90, p <.05, ηp^2^=.014].

**Figure 2:**
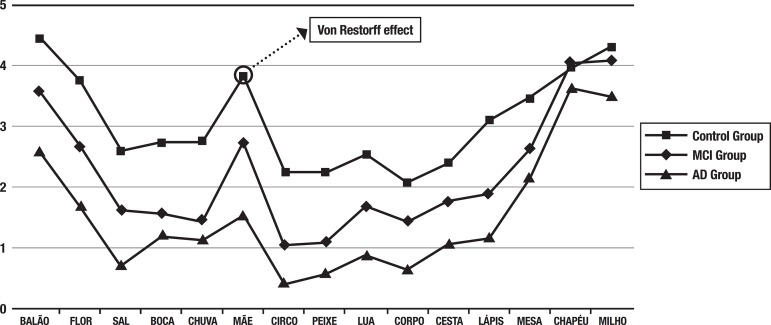
Serial position curve for all three groups. Original words (in Portuguese) from the RAVLT are shown.

Figure 2 shows the Von Restorff effect, giving the RAVLT curve a W-shape.

## DISCUSSION

We demonstrated that performance on the Brazilian version of the RAVLT is increasingly impaired when administered to patients with MCI and AD, respectively, relative to matched controls, corroborating reports for other samples from different cultures and backgrounds.^36^^,^ 37 Notwithstanding, sensitivity and other psychometric properties of the RAVLT are beyond the scope of this manuscript and will be addressed in a separate paper. In the present study, we aimed to demonstrate that a specific word with emotional weight (mother) included in the latest version of the list was associated with the Von Restorff effect, changing the traditional U-shape of the serial position recall curve into a W-shape. We also demonstrated that the effect was present in healthy elderly - a fact that has been previously the subject of debate.

Our results support those of previous studies,^7^^,^ 11 ^,^ 12 which also found that the isolation effect influences memory recall in smaller samples of older adults. We hold that the isolation effect displayed by older adults can be observed across different methodologies used to investigate this phenomenon, regardless of whether the isolated word is emotionally distinct (as in this study), or differs semantically^11^ or physically from the remaining items.^7^^,^ 12

In addition, we also investigated the presence of the Von Restorff effect in clinical samples with memory impairment (amnestic MCI and AD patients). Only one study studied this effect in an AD population.^12^ However, our results contrast with the previous study assessing the effect in the AD population, which suggested that a floor effect would account for the absence of the Von Restorff effect in the group.^12^ One explanation for the disparate results found in the present study might be the differences in the methodologies applied by each study. In our study, a single 15-word list was aurally presented five times to each subject, while the cited study presented several 25-item isolation lists visually. As noted earlier, presenting several lists may be less ideal for studying older populations due to the potential for differential effects of interference.^38^ This can hold especially true for memory-impaired individuals.

To our knowledge, this is the first paper to assess the presence and magnitude of this effect on these three clinical samples altogether. In the present study, we demonstrated that this effect did not disappear when memory deficits increased in mild (MCI) and severe cases (AD), although this was more subdued in the latter group.

In general, AD is a progressive disease caused by changes in the medial temporal lobe (MTL) including the amygdala, thought to play a crucial role in emotional memory.^39^ In fact, there is evidence that the hippocampal system plays a critical role in Von Restorff effects.^8^ Supporting these findings, there is also evidence from other neuroimaging studies showing reduced hippocampal volume and cortical thickness in MCI and AD patients.^40^^,^ 41 In addition, another study observed activation of the medial temporal lobe and medial frontal regions in both the control group and MCI group during an emotional memory paradigm, although this activation was not observed in the AD group.^42^

Although not all MCI patients progress to AD, it is recognized as a pre-clinical stage of AD^43^ and, while MCI due to AD mainly presents with MTL atrophy (while other regions are relatively preserved), as AD progresses, atrophy spreads to the frontal lobe.^44^ Many studies also show that the frontal cortex plays an important role in the Von Restorff effect.^13^^,^ 45 ^,^ 46 More specifically, previous studies have shown that orbitofrontal and lateral prefrontal regions might modulate the isolation effect.^45^ Furthermore, medial prefrontal cortex (PFC) and lateral PFC have been implicated in the functional link between specialized regions in AD patients.^47^

Thus, neuroanatomical findings suggest that reduced Von Restorff in AD may have several neuroanatomical roots. Regarding the results observed in the present study, one could argue that changes in the MTL or PFC may explain these, or that a combination of both may account for the findings. Nevertheless, it is not our objective here to postulate why the reduction in the Von Restorff effect is more prominent in the AD group, yet remains relatively preserved in MCI group. However, in a more general view, it seems clear that disease progression causes impairment in the memory for distinct stimulus.

Finally, focusing on clinical applications, future studies should investigate the number of times the Von Restorff effect word (mother) is repeated during the learning phase (compared to other words from the list) in diverse clinical situations (i.e., different amnestic syndromes), especially when serial position effects are the focus of analyses.
